# Antithrombin during veno-venous extracorporeal membrane oxygenation with heparin anticoagulation: A single-center cohort study

**DOI:** 10.1177/02676591241258048

**Published:** 2024-06-04

**Authors:** Bethany A Hileman, Gennaro Martucci, Nicolò Rizzitello, Giovanna Occhipinti, Matteo Rossetti, Fabio Tuzzolino, Roberto Lorusso, Mauro Panigada, Kenichi Tanaka, Antonio Arcadipane, Giovanna Panarello

**Affiliations:** 1 12317 University of Pittsburgh School of Medicine, Pittsburgh, PA, USA; 2 Department of Anesthesia and Intensive Care, 18326 Istituto Mediterraneo per i Trapianti e Terapie ad alta Specializzazione (IRCCS-ISMETT), Palermo, Italy; 3 657897 University of Pittsburgh Medical Center Italy, Palermo, Italy; 4 Statistics and Data Management Services, 18326 Istituto Mediterraneo per i Trapianti e Terapie ad alta Specializzazione (IRCCS-ISMETT), Palermo, Italy; 5 Cardiothoracic Surgery Department, Maastricht University Medical Center and Cardiovascular Research Institute Maastricht, 82246 Maastricht University, Maastricht, Netherlands; 6 Department of Anesthesia and Critical Care, Fondazione IRCCS Ca’ Granda - Ospedale Maggiore Policlinico, Milan, Italy; 7 The University of Oklahoma Health Sciences Center, 12308 University of Oklahoma, Oklahoma City, OK, USA

**Keywords:** antithrombin, heparin, anticoagulation, bleeding, extracorporeal membrane oxygenation

## Abstract

**Introduction:**

Antithrombin (AT) is a natural anticoagulant essential to enhancing the unfractionated heparin (UFH) anticoagulant effect. Its supplementation in the management of UFH-based anticoagulation during veno-venous extracorporeal membrane oxygenation (VV ECMO) has a strong pathophysiological rationale.

**Methods:**

This is a single-center, retrospective cohort study of adult VV ECMO patients with anticoagulation maintained by UFH targeting an activated partial thromboplastin time (aPTT) of 40–50 s and AT activity >80%. We compare anticoagulation management and survival outcomes between AT subpopulations, defined by a threshold AT activity ≥80%. Linear and logistic regression analyses were used to evaluate the variation in AT activity and its association with ICU survival.

**Results:**

In 244 patients enrolled from 2009 to 2022, anticoagulation was maintained by a median heparin dose of 11.4 IU/kg/h [IQR: 8.2–14.7] with a mean aPTT of 46.1 s (±7.3) and AT activity of 88.9% (±17.0). A lower mean aPTT, higher dose of UFH and shorter fraction of time without UFH were associated with higher AT activity (*p* < .01). Higher AT activity showed a consistent association with ICU survival (for 10% increase of AT, odds ratio for ICU mortality: 0.95; 95% CI 0.93–0.97; *p* value <.01).

**Conclusions:**

There is a positive association between AT activity and UFH requirements but no significant difference in the rate of bleeding events. A higher mean AT during VV ECMO was associated with ICU survival. Future studies are needed to differentiate between exogenously supplemented versus endogenous AT effect.

## Introduction

During veno-venous extracorporeal membrane oxygenation (VV ECMO), continuous contact of blood with the extracorporeal circuit causes systemic activation of inflammatory and coagulation pathways, increasing the risk for thrombosis and consumption of coagulation factors.^1^ Despite the technological advancement of coating methods for cannulas and circuits, unfractionated heparin (UFH) is still the most common anticoagulant used as a continuous infusion in adult and pediatric ECMO settings.^2,3^ UFH exerts its pharmacodynamic effect by binding to antithrombin (AT) and causing >1000-fold enhancement in AT inhibition predominately of thrombin (factor IIa) and factor Xa, as well as plasmin, factor IXa, factor XIa, and factor XIIa.^4^

Current guidelines for heparin-based anticoagulation protocols recommend monitoring with indirect measures of the heparin effect rather than targeting a standard heparin dose.^5^ However, there is a lack of consensus and evidence for the best test to measure the heparin effect and the goal that should be targeted.^6,7^ Monitoring anticoagulation with heparin is further complicated by the dependence of UFH on an unknown minimal level of AT and influence from other blood components.

AT activity is only monitored in 48.7% and routinely supplemented in 38.1% of ECMO centers.^8^ The indication for supplementing AT among the centers that reported AT supplementation was most commonly to help reach and/or maintain the anticoagulation goal. Similarly, the decision not to supplement AT among the centers that reported no AT supplementation was explained by a lower degree of difficulty in reaching and/or maintaining the anticoagulation goal.

AT also has a role as an inflammatory response molecule.^9^ Previous studies suggest an association between AT activity and ICU survival.^10^ Additionally, AT is inversely associated with markers of inflammation, whether by endogenous activity or exogenously supplemented.^11^ Taken together, the survival benefit and pleiotropic effects of AT point towards the potential use of AT monitoring and supplementation to stabilize the coagulation/anticoagulation balance. AT supplementation has not been shown to reduce the heparin requirement in VV ECMO,^12^ but the requirement is already low relative to that used in ECMO for cardiopulmonary support. The high incidence of bleeding and thrombotic events with a strong association to hospital mortality ultimately emphasizes the need for further investigation to resolve the many uncertainties surrounding anticoagulation during VV ECMO.^13^

The present study aims to describe AT activity among adult VV ECMO patients treated on a heparin anticoagulation protocol, to identify factors positively associated with variation in AT activity, and to explore the hypothesis of a positive association between higher AT activity and ICU survival.

## Methods

This single-center, retrospective cohort study was approved by the Institutional Review Board of ISMETT (IRRB/34/19). The study population includes consecutive patients supported on VV ECMO for respiratory failure between November 2009 and June 2022. Exclusions were based on the following criteria: less than 18 years old, diagnosis of end stage respiratory failure, any veno-arterial configuration (either central or peripheral cannulation) during the ICU stay (also prior to VV ECMO start).

### Data collection and rationale of the study

All data were retrieved from the electronic medical record system by automated extraction. Baseline data describe the demographic and patient characteristics prior to initiation of ECMO support, including hospital length of stay (LOS), intensive care unit (ICU) LOS, and duration of mechanical ventilation (MV). Baseline data also report the Simplified Acute Physiology Score (SAPS) II, Murray score, Sequential Organ Failure Assessment (SOFA), and Respiratory Extracorporeal Membrane Oxygenation Survival Prediction (RESP) scores.

The primary aim of the study was to describe the real-practice implementation of an anticoagulation protocol based on UFH infusion and the potential administration of AT. The application of the protocol is described by daily AT activity level, dosage, and number of administrations. To substantiate this goal, the factors (at baseline and during ECMO management) associated with AT variations were explored, and the safety of AT administration was evaluated by the rate of bleeding complications. The secondary aim was to delve into the association between the plasma level of AT activity and ICU survival, considering that AT has been found to be pivotal in host defense and inflammation.

### Anticoagulation protocol, measurement, and administration of antithrombin

Anticoagulation was maintained with UFH, which is the principal method adopted worldwide in VV ECMO (starting conventionally at 8 units/kg/h).^3,10,14^ Monitoring involved aPTT being checked every 4 h (targeted to a range of 40 to 50 s) and AT activity once daily and supplemented if the value was <80%, according to the internal clinical pharmacy protocol. At our institute over the years, only human-derived AT has been available. AT activity levels were measured using the INNOVANCE Antithrombin assay with the Sysmex CS-5100 coagulation analyzer system (Siemens Healthcare GmbH, Erlangen, Germany). Evaluation in a multicenter study, with a comparison of this factor Xa-based assay to the well-established factor IIa and other factor Xa-based assays, confirmed its precision and reliability in the detection of AT deficiency.^15^ The final decision to supplement AT was taken by the attending physician based on the perceived benefit and by the availability of the drug. During bleeding events, UFH was temporarily suspended and PRBC transfusions were given to maintain hematocrit between 24% and 30%. Platelet transfusions were usually given if platelet counts dropped below 50,000 per mL, but the decision to transfuse was taken on a case-by-case evaluation. UFH was the only anticoagulant drug available at our institute. Consequently, in the case of bleeding and coagulation impairment, the practice was to stop anticoagulation until recovery from the bleeding episode or coagulation resettling.

### Statistical analyses

Categorical variables are reported as frequency and percentage. Continuous variables are reported as mean and standard deviation or median and interquartile range (25^th^ to 75^th^ percentile; IQR).

AT was calculated as the average daily level during VV ECMO support of individual patients, and then two subpopulations were defined by differentiating the average AT activity level according to the lower reference interval of our laboratory: the low AT group had AT activity <80% and the high AT group had AT activity ≥80%. Subpopulation comparisons were assessed by a chi-square test for categorical variables and a 2-sample *t* test for continuous variables, using the pooled method for equal variances and the Satterthwaite approximation for unequal variances. To highlight if extreme deviations from the normal range values (80%–100%) in AT have any impact on the outcomes, the association between ICU mortality and AT levels as well for inflammation biomarkers (WBC, CRP, and procalcitonin) were also considered separately according to average AT level for patients divided into three groups (below 80%, between 80 and 100%, and above 100%).

A linear regression analysis was applied to evaluate correlations between average daily AT activity and continuous variables. The purpose of this analysis was to assess the behavior of AT activity with respect to continuous explanatory variables and understand how they could act together with respect to ICU discharge.

After the preliminary regression analysis, a penalized logistic model with LASSO selection was used to evaluate the relevance of the association between the covariates and ICU discharge status.^16,17^ The LASSO idea is quite general and can be applied in a variety of statistical models. The logistic LASSO model is a shrinkage method to select from a large and potentially multicollinear set of variables, resulting in a more relevant and interpretable set of predictors. LASSO performs a continuous shrinking operation, minimizing regression coefficients in order to reduce the likelihood of overfitting. However, the technique is computed so as to shrink the sum of the absolute value of regression coefficients, forcing and producing coefficients that are exactly 0, thus selecting for the nonzero variables to remain in the model. To handle the missing data problem, multiple imputation was used and a different dataset was used to compute logistic LASSO regression.

Odds ratios are reported with 95% confidence intervals. The determination of statistical significance was based on a *p* value threshold of <0.05. Data analyses were completed using the SAS 9.4 (SAS Institute Inc., Cary, North Carolina, U.S.A.) and R 4.0.3 statistical software (R Core Team (2022).

## Results

During the study period, a total of 288 patients received ECMO support. Some patients were excluded for the following reasons: six were less than 18 years old, 25 were diagnosed with end-stage respiratory failure, and 13 with veno-arterial configuration for intraoperative support and cardiopulmonary failure. A total of 244 patients were included in the study. Baseline data and subpopulation comparisons are summarized in Table 1.

**Table 1.** Baseline data and antithrombin activity subpopulation comparisons.

Variable	Total	AT activity <80%	AT activity ≥80%	*p* value
	*N* = 244	*N* = 66	*N* = 178	
Baseline characteristics				
Age, years	46.3 (12.4)	48.7 (11.6)	45.4 (12.6)	.07
Male gender, *N* (%)	190 (77.9%)	49 (74.2%)	141 (79.2%)	.41
BMI, kg/m^2^	29.0 (6.0)	27.3 (4.8)	29.6 (6.2)	**<.01**
SAPS II	38.4 (10.5)	42.0 (12.0)	37.0 (9.6)	**<.01**
Murray score	3.3 (0.61)	3.3 (0.6)	3.3 (0.6)	.66
SOFA score	8.0 [6.0–10.0]	9.0 [7.0–11.0]	8.0 [6.0–10.0]	**<.01**
RESP score	1.0 [–1.0–2.0]	0.0 [–1.0–1.0]	1.0 [–1.0–3.0]	**<.01**
Hospital LOS pre-ECMO, days	7.0 [2.0–14.0]	9.0 [4.0–18.0]	7.0 [2.0–13.0]	.11
ICU LOS pre-ECMO, days	5.0 [2.0–10.0]	6.0 [2.0–12.0]	3.2 [2.0–9.0]	.07
MV duration pre-ECMO, days	5.0 [2.0–9.0]	7.0 [3.0–12.0]	3.2 [2.0–8.0]	**.01**
COVID-19 diagnosis, *N* (%)	87 (35.7%)	23 (34.9%)	64 (36.0%)	.87
ECMO management & complications				
Femoro-jugular, *N* (%)	172 (72.9%)	42 (66.7%)	130 (75.1%)	.20
Femoro-femoral, *N* (%)	64 (27.1%)	21 (33.3%)	43 (24.9%)	.20
Mean HTC, %	28.1 (3.9)	28.2 (3.9)	28.1 (3.9)	.78
PLT nadir, µL	66.0 [35.0–111.0]	45.5 [15.0–80.5]	73.0 [44.0–115.0]	**.04**
Mean aPTT, seconds	46.1 (7.3)	49.7 (10.5)	44.9 (5.4)	**<.01**
Mean WBC, 10^3^/µL	13.9 [10.5–17.8]	14.1 [9.4–20.8]	13.8 [10.8–17.2]	.56
Mean CRP, mg/dL	132.9 [98.9–183.0]	134.3 [91.7–206.6]	132.2 [101.0–173.8]	.11
Procalcitonin, ng/mL	3.3 [1.2–10.5]	4.5 [1.3–10.5]	2.0 [1.0–10.3]	.33
Mean AT activity, %	88.9 (17.0)	68.4 (15.3)	95.9 (10.8)	**<.01**
Total AT dose, IU	1857.1 [1500.0–2000.0]	2000.0 [1707.2–2017.0]	1789.5 [1375.0–2000.0]	**.05**
Daily AT dose, IU/kg/day	0.9 (0.4–1.8)	1.5 (0.5–2.5)	0.7 (0.4–1.5)	**.03**
AT frequency, *N*	4.0 [2.0–11.0]	6.5 [2.0–16.0]	4.0 [1.0–10.0]	**.04**
Mean heparin dose, IU/kg/h	11.4 [8.2–14.7]	9.7 [5.4–14.3]	11.5 [8.7–14.7]	**<.01**
Percent of heparin-free hours	21.4 [7.1–49.2]	33.3 [5.3–72.1]	17.0 [7.2–40.6]	**<.01**
Total PRBC, mL	2166.0 [820.0–5070.0]	9.0 [5.0–20.0]	6.0 [2.0–15.0]	.38
Mean daily PRBC, mL	102.2 [55.0–176.0]	145.0 [72.0–266.8]	94.3 [50.0–155.0]	**<.01**
FFP Transfused, *N* (%)	45 (18.6%)	16 (25.0%)	29 (16.3%)	.12
PLT transfused, *N* (%)	74 (30.5%)	26 (40.0%)	48 (27.0%)	**.04**
≥1 bleeding event, *N*	93 (44.3%)	28 (49.1%)	65 (42.5%)	.46
ECMO duration, days	19.0 [10.5–40.5]	16.0 [7.0–33.0]	21.0 [11.0–43.0]	.30
Clinical outcomes				
ECMO survival, *N* (%)	168 (68.9%)	29 (43.9%)	139 (78.1%)	**<.01**
ICU Survival, *N* (%)	154 (63.1%)	25 (37.9%)	129 (72.5%)	**<.01**

COVID-19, coronavirus disease 2019; BMI, body mass index; SAPS, simplified acute physiology score; SOFA, sequential organ failure assessment; RESP, respiratory ECMO survival prediction; LOS, length of stay; ECMO, extracorporeal membrane oxygenation; ICU intensive care unit; MV, mechanical ventilation; HTC, hematocrit; PLT, platelet; aPTT, activated partial thromboplastin time; WBC, white blood cell count; CRP, C-reactive protein; AT, antithrombin, PRBC, packed red blood cells; FFP, fresh frozen plasma Eight patients were cannulated with a double lumen catheter (Avalon).

Bold text indicates statistically significant *p* values.

**Figure 1.**
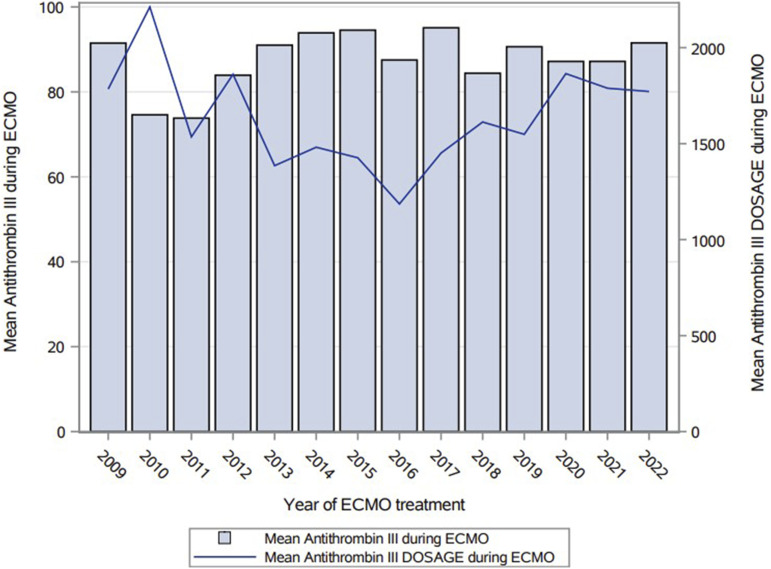
Mean antithrombin activity level per year of ECMO treatment.

Overall, anticoagulation was achieved by a median UFH dose of 11.4 IU/kg/h [IQR: 8.2–14.7] with a mean aPTT of 46.1 s (±7.3) and an average AT activity level of 88.9% (±17.0) over a median ECMO duration of 19.0 days [10.5–40.5]. Sixty-six patients (27%) had an average AT value <80%, 128 (53%) were in the range between 80 and 100, while 50 (20%) had an average AT higher than 100%. The average AT activity level was stable over the years, *p* value = .1724 (Figure 1).

AT was administered at least once in 215 (88%) patients, while 29 patients did not receive any AT supplementation during the ECMO stay. For those who did not receive any AT, the mean AT plasma level of activity was 97 (±28), while for patients with supplementation it was 88 (±15). The median dose of AT administered throughout the duration of the ECMO support was 1857 IU [1500–2000], while considering the values administered according to weight and independently from the ECMO duration the dose was 0.9 IU/kg/day (0.4–1.8). Anticoagulation among patients in the high AT subpopulation was achieved by a significantly higher heparin dose (11.5 vs 9.7 IU/kg/h; *p* < .01) with a lower aPTT (44.9 vs 49.7 s; *p* < .01). To maintain the AT activity target, the mean dose of AT administered in the high AT subpopulation was significantly lower (0.7 vs 1.5 IU/kg/day; *p* = .03). The frequency of AT administration was significantly lower in the high AT subpopulation (4.0 vs 6.5; *p* = .04) without a difference in ECMO duration. Additionally, the high AT subpopulation demonstrated a higher platelet count nadir (*p* = .04), lower rate of PLT transfusions (27.0% vs 40.0%; *p* = .04), and smaller total amount of PLT transfused (*p* = .03). Looking at the principal causes of ARDS, the high AT versus the low AT group did not show a significant difference: bacterial pneumonia *n* = 38 (16%) versus *n* = 13 (5%), viral pneumonia *n* = 53 (22%) versus *n* = 14 (6%), COVID-19 *n* = 64 (26%) versus 23 (9%), trauma *n* = 13 (5%) versus *n* = 5 (2%), and graft failure *n* = 4 (2%) versus *n* = 6 (2%).

### Factors associated with average AT activity during VV ECMO

The covariates associated with variation in the average AT activity level during VV ECMO are presented in Table 2.

**Table 2.** Factors associated with variation in mean daily antithrombin activity during VV ECMO.

	Parameter estimate	Standard error	*p* value
Baseline characteristics			
Age, 10 years	−1.6	0.9	.08
BMI, 5 kg/m^2^	2.7	0.9	<.01
SAPS II, 10 points increase	−4.5	1.11	<.01
SOFA score, 2 points increase	−2	0.9	.02
RESP score	1.72	0.4	<.01
MV duration pre-ECMO, 7 days	−1.8	1.1	.09
ECMO management & complications			
PLT nadir, 10.000 µL	0.1	0.01	<.01
Mean aPTT, 10 s	−5.8	1.47	<.01
Mean WBC, 10^3^/µL	−0.08	0.15	.59
Mean CRP, mg/dL	−6	1.5	<.01
Mean heparin dose, IU/kg/h	0.61	0.22	<.01
Daily AT dose, IU/kg/day	−2	0.6	<.01
UFH-free hours, 10% increase	−1.3	0.4	<.01
Total PRBC, 1000 mL	−0.2	0.03	<.01
Total FFP, 1000 mL	−3.9	1.8	.03
Total PLT, 500 mL	−1.5	0.5	<.01
ECMO duration, 10 days	0.1	0.3	.73

Among the baseline characteristics, higher average AT activity levels were significantly associated with a higher BMI and lower severity at cannulation (lower SAPS II, lower SOFA score, and higher RESP score). Regarding management during VV ECMO, there was a significant association between higher average AT activity and higher platelet count nadir (*p* < .01), lower mean aPTT (*p* < .01), lower mean CRP (*p* < .01), higher mean heparin dose (*p* < .01), smaller percentage of time without heparin (*p* < .01), lower total amount of FFP transfused (*p* = .03), and lower total amount of PLT transfused (*p* < .01). The correlation between AT and biomarkers related to inflammation and bacterial infection (CRP and procalcitonin) was negative: Pearson correlation coefficient for CRP −0.27 *p* < .01, for procalcitonin −0.27 *p* = .05. Considering patients with different average AT during ECMO (<80, 80–100 and >100) we did not find significant differences in the associations with available parameters for inflammation (WBC, CRP, procalcitonin), although the bio-markers of inflammation showed higher values in the group with AT <80.

### AT activity and ICU survival after VV ECMO

Regarding clinical outcomes, the population with an average AT activity level greater than 80% had higher rates of ECMO survival (78.1% vs 43.9%; *p* < .01) and ICU survival (72.5% vs 37.9%; *p* < .01) (Table 1). This increase in survival was even more evident in the group with an average AT level >100: 86% for AT >100, 67% for patients in the range 80–100, 38% for patients with AT <80, *p* value at ANOVA <0.01. Among patients without supplementation, 21 out of 29 (72%) survived at ICU discharge, compared to 133 out of 215 (62%) patients with at least one AT administration, *p* value = .13. An increase of 10% activity in the average AT activity level during VV ECMO (adjusted for the duration of ECMO) showed a consistent association with a reduction in ICU mortality: odds ratio 0.95; 95% confidence limits 0.93–0.97; *p* value <.01. Considering the principal causes of ARDS, higher survival rates were seen in cases of bacterial pneumonia (67%), trauma (67%) and viral pneumonia (76%), while a lower rate was achieved for COVID-19 diagnosis (52%). The significance and magnitude of this association was maintained upon adding adjustments for the majority of the other variables described in Table 3. Considering other inflammatory factors available (WBC, CRP, and procalcitonin), procalcitonin was the only covariate that made such an association non-significant.

**Table 3.** Odds ratios for ICU mortality according to AT levels in a bivariate analysis adjusted for covariates.

Adjusted variable	Odds ratio	95% confidence interval	*p* value
**Baseline characteristics**			
Age, years	0.95	0.93–0.97	<.01
Male gender	0.95	0.93–0.97	<.01
BMI, kg/m^2^	0.95	0.93–0.97	<.01
SAPS II	0.96	0.94–0.98	<.01
SOFA score	0.95	0.93–0.98	<.01
RESP score	0.95	0.93–0.97	<.01
MV duration pre-ECMO, days	0.95	0.93–0.98	<.01
COVID-19 diagnosis	0.95	0.93–0.97	<.01
ECMO management & complications			
Mean PLT nadir, µL	0.96	0.94–0.99	<.01
Mean aPTT, seconds	0.95	0.93–0.98	<.01
Mean WBC, 10^3^/µL	0.95	0.93–0.97	<.01
Mean CRP, mg/dL	0.95	0.93–0.98	<.01
Mean procalcitonin	0.97	0.93–1.01	.10
Mean heparin dose, IU/kg/h	0.96	0.94–0.98	<.01
Percent of heparin-free hours	0.96	0.94–0.98	<.01
Total PRBC, mL	0.95	0.93–0.97	<.01
Total FFP, mL	0.95	0.93–0.97	<.01
Total PLT, mL	0.96	0.94–0.98	<.01
ECMO duration, days	0.95	0.93–0.97	<.01

**Figure 2.**
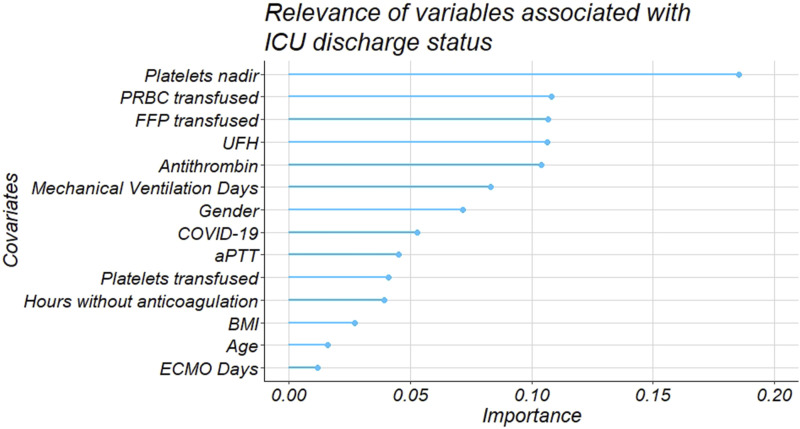
LASSO procedure for multiple penalized logistic regression model.

The relevance of average AT activity level on the ICU mortality outcome was confirmed after a penalized multiple logistic regression with LASSO feature selection for all the covariates (Figure 2).

## Discussion

The present retrospective, single-center study describes the use of AT as a component of a heparin-based protocol for anticoagulation during VV ECMO, exploring two large topics related to AT: the impact on the coagulation profile and the relation with the patient’s severity and likely the outcome. The results report a moderate association of higher AT activity with lower average aPTT, higher dose of heparin, and shorter fraction of time without heparin during the ECMO stay. Moreover, the higher AT level during ECMO was associated with lower pre-ECMO severity scores and was associated consistently with improved ICU survival.

UFH is dependent on AT to achieve anticoagulation, but the enhancement of AT activity by heparin also enhances the consumption of AT, leading to acquired AT deficiency.^18,19^ Therefore, the theoretical basis for AT supplementation in heparin-based anticoagulation protocols is that correction, or prevention, of acquired AT deficiency would reduce UFH requirements to maintain a target anticoagulation.^18^ Retrospective studies in pediatric populations are inconsistent about the effect of AT supplementation on the heparin requirements.^20–23^ A randomized controlled trial in an adult population similarly found no significant reduction of heparin requirements with AT supplementation.^12^ A recent large observational study illustrated how the AT measurement is sporadic in UFH-based protocols and, as a consequence, its administration is rare.^14^ Our results show first, that AT was consistently consumed during ECMO as indicated by 27% of patients with lower average values despite active supplementation; second, UFH dose was associated with a higher average AT activity.

Current guidelines recommend AT supplementation to maintain AT activity in the range 80%–120% or in the setting of circuit clotting despite normal-high heparin dosing, depending on the availability of an AT assay.^5^ However, there is neither a standard dose of heparin nor a perfect laboratory test to monitor heparin activity for anticoagulation in ECMO.^5,7,24^ Similarly, the minimum AT activity level required for the heparin effect remains undefined.^18^ Therefore, when and how much AT should be supplemented is largely unknown, as well as the way of administration. We reported the administration of boluses repeated during the ECMO stay, corresponding to a dose about 1 IU/kg/day. This is also related to the vast field of AT measurement. Our method is considered quite advanced since it is able to detect AT deficiencies in other deficiency coagulopathies, but the discrepancy among different assays is still debated.^25^

Our anticoagulation protocol targeted a low aPTT goal, 40–50 s, and normal-high AT activity, greater than 80%. We identified a moderate correlation of higher average AT activity with decreased aPTT. However, contrary to our expectations, higher AT activity also showed a moderate correlation with increased heparin dose. Therefore, it is unclear whether AT is a confounding factor in the relationship between heparin dose and reduced aPTT. Despite lower average AT activity, the low AT subpopulation demonstrated reduced heparin requirements. We also observed significantly more frequent and higher doses of AT supplementation among the low AT subpopulation. Therefore, irrespective of maintaining elevated AT activity, AT supplementation might be associated with a reduction in UFH requirement. Generally, in the context of a highly imbalanced coagulation status, spontaneous higher levels of AT activity might be associated with a more stable coagulation profile with fewer anticoagulant requirements. However the picture is also likely complicated by the increased thrombin generation in the case of low AT levels: Tsuchida et al. demonstrated a higher thrombin generation capacity when the AT activity is decreased below 50%.^26^ This might raise the hypothesis that AT contributes to the balance of the coagulation system and plays a role in the crossroads between different biological pathways. Clearly, the picture of coagulation is complicated by the presence of the extracorporeal circuit which invariantly creates a disseminated coagulopathy (DIC)–like profile, thus there should be consideration for factors and coagulation processes with extensive monitoring of fibrinogen, D-dimers, LDH, hemolysis, and functional coagulopathy.^27,28^ To conclude on this issue, coagulation is determined by several factors and processes, so a personalized approach is probably warranted and should be based on studies of primary and secondary hemostasis^29–31^; AT may reasonably be measured to understand the balance of the anticoagulation process mainly during UFH-based protocols.

The second relevant topic related to AT during VV ECMO is the patient’s severity and outcomes. Coagulation profile scores have been linked to mortality, although the pathophysiological mechanisms are not overtly understood.^32^ Considering AT, it has coagulation-dependent and independent anti-inflammatory properties, in addition to its anticoagulant effects.^4,33^ We found that higher AT activity was correlated with lower CRP but not WBC. Additionally, there were no significant differences in CRP or WBC between the AT subpopulations averaged across the duration of ECMO, but in the group with AT <80 the inflammation bio-markers were higher although without statistically significative differences. This lack of differences may be explained by the heterogeneity of indications for ECMO in both groups as well as for the high amount of confounding factors.

The level of inflammation is probably one of the more relevant biologic features of severity. Actually, the various severity scores adopted were all associated with a higher level of AT and higher probability of survival, both considering cumulative scores like SAPS II, SOFA and RESP scores, and the length of mechanical ventilation prior to ECMO, another relevant feature of prediction in ECMO weaning (and consequently mortality). A recent study showed that plasma levels of AT (either endogenous or exogenous) were negatively associated with inflammation (cytokines) and suggested that the administration of antithrombin may lead to a more rapid decrease over time of cytokines (namely IL-6, IL-1β, TNF-⍺ and Pro-ADM), however causal effect is still to be demonstrated.^11,34–36^ Accordingly, the high AT subpopulation showed a significantly lower SAPS II and SOFA score and a higher RESP score. These findings demonstrate reduced severity among the high AT group. In addition to the aforementioned association between severity and AT, we previously identified an inverse association between AT activity and ICU mortality.^10^ We now provide further support for this association by demonstrating improved ICU survival among the patients maintained at an AT activity above the threshold of 80%, and this association remains relevant despite varying ECMO durations and several additional adjustments. Moreover, this increase in survival shows linear progression and is confirmed also for average AT values higher than 100%. This contrasts with previous data from patients with sepsis but might be linked to the different origin of the coagulation imbalance.^37^

The previous considerations and results presented in this study explain, at least in part, the highly variable pattern of AT monitoring and supplementation during ECMO.^8^ This variability in practice is consistent with the different practices among centers for most aspects of hematological management in ECMO.^38,39^ The higher AT subgroup received less frequent and smaller AT supplementations, suggesting that higher AT activity is more likely to be achieved by spontaneous production (or less consumption) in the setting of coagulation activation, as suggested by the higher values in patients without supplementation. Therefore, higher AT activity achieved with exogenous AT supplementation might not have the same survival benefit as higher endogenous AT activity. On the other hand, early AT administration might contribute to hemostatic stability and overall better clinical status, but this hypothesis should still undergo prospective studies.

Finally, despite the lack of reduction of heparin requirements, AT supplementation to target high AT activity levels appears to be safe since no significant difference in the rate of bleeding events between the high and low AT subpopulations occurred. A randomized controlled trial also found no difference in bleeding or thrombotic events.^12^ Therefore, future investigations and prospective trials in large cohorts can still be considered.

Our study has several limitations. First, this is a single-center study with cases collected over several years. Second, the mixed cases might include different causes of acute respiratory failure with a different impact on the coagulation status. Third, we are unable to distinguish the effects of endogenous AT activity from exogenously supplemented AT activity. Fourth, AT monitoring is not part of the regular coagulation profile in the general ICU, and considering that 99% of our patients on ECMO are transferred from regional referral hospitals, consequently data were not available before ECMO cannulation and, despite assuming that during ECMO AT undergoes substantial changes, we cannot exclude congenital differences in AT production. Fifth, due to lack of request during the proposal for automated data extraction we do not currently have available the nadir of AT during the ECMO stay. Finally, many unmeasured confounders may affect the interpretation of our results.

## Conclusions

Higher AT activity averaged across the duration of ECMO was associated with improved ICU survival. The dose and frequency of AT supplementation was inversely associated with AT activity. Although AT activity showed a strong positive correlation with higher heparin requirements, higher average AT activity did not show a significant difference in the rate of bleeding events. Therefore, future studies should compare the effects of higher endogenous AT activity with exogenously supplemented AT activity to differentiate between their contributions to the survival benefit.
